# Computation of Conformational Coupling in Allosteric Proteins

**DOI:** 10.1371/journal.pcbi.1000484

**Published:** 2009-08-28

**Authors:** Brian A. Kidd, David Baker, Wendy E. Thomas

**Affiliations:** 1 Department of Bioengineering, University of Washington, Seattle, Washington, United States of America; 2 Department of Biochemistry, University of Washington, Seattle, Washington, United States of America; 3 Howard Hughes Medical Institute, Seattle, Washington, United States of America; Weizmann Institute of Science, Israel

## Abstract

In allosteric regulation, an effector molecule binding a protein at one site induces conformational changes, which alter structure and function at a distant active site. Two key challenges in the computational modeling of allostery are the prediction of the structure of one allosteric state starting from the structure of the other, and elucidating the mechanisms underlying the conformational coupling of the effector and active sites. Here we approach these two challenges using the Rosetta high-resolution structure prediction methodology. We find that the method can recapitulate the relaxation of effector-bound forms of single domain allosteric proteins into the corresponding ligand-free states, particularly when sampling is focused on regions known to change conformation most significantly. Analysis of the coupling between contacting pairs of residues in large ensembles of conformations spread throughout the landscape between and around the two allosteric states suggests that the transitions are built up from blocks of tightly coupled interacting sets of residues that are more loosely coupled to one another.

## Author Summary

A common means of biological regulation is allostery, in which an effector molecule binds to one site on a protein and induces a conformational change which changes activity at a distant active site. Frequently high resolution structures are determined for one state of an allosteric protein but not the other. To probe the allosteric conformational changes in such cases, we describe a computational method for predicting the structure of one allosteric state of a protein starting with knowledge of another. Our method also provides a detailed map of the free energy landscape traversed in an allosteric transition and reveals the coupling between interacting residue pairs that underlies the transition.

## Introduction

Allosteric transitions, in which binding of an effector molecule to one site of a protein is coupled to a conformational change at a distant site, are fundamental to biological regulation. Although the first models were proposed more than 40 years ago [1],[2], developing a mechanistic understanding of allostery continues to be an active and vigorous area of research [3],[4]. For a small number of allosteric proteins, X-ray crystal structures of ligand bound and ligand free states have illuminated the structural transitions underlying allostery [5]–[10]. However, the small number and static nature of these structures present several important challenges for structural biology that may be approached using computational methods.

First, it may be possible to *predict* the structure of the one allosteric state starting from the structure of the other state. Meeting this challenge requires both an efficient method for conformational sampling in the neighborhood of the starting state and a sufficiently accurate energy function. Predicting the bound state from the unbound state is more challenging because it requires solving both the docking problem and the allosteric conformational change problem simultaneously. Predicting the unbound state from the bound structure is more straightforward and hence is a natural first step toward addressing the general prediction challenge. A successful approach would be extremely useful for predicting the conformational changes that occur in an allosteric protein for which only the structure of the bound state is available.

A second challenge is to determine the *mechanisms* controlling allosteric regulation by identifying how individual residues are involved in allosteric transitions. Normal mode analysis of elastic network models [11]–[13], a nonlinear elastic model [14], network modeling of contact rearrangements [15], and statistical coupling of local unfolding [16],[17] have all been applied to protein structures to investigate mechanisms of conformational switching. These methods work best for identifying global motions, geometrical differences, or residue stability. NMR and other data suggest that most allosteric proteins are essentially two state systems, with bound and unbound states, but not intermediate states, populated at equilibrium [18],[19]. Since states intermediate between the observed bound and unbound states are higher in free energy and cannot be readily observed experimentally, it is difficult to map the free energy landscape between the two states using experimental methods. One computational approach has been to use a multiple basin model to map the free energy landscape and approximate the transition between states [20],[21]. However, this method only considers Cα atoms and utilizes knowledge of the structural end points as references in the potential function. Insight into residue couplings has come from studies of evolutionary covariance [22]–[24], but this method can only be applied to systems with a large and diverse set of sequences. All-atom molecular dynamics simulations [25]–[28] can show residue couplings in great detail, but only when conformational transitions occur in the nanosecond timescale.

The Rosetta high-resolution structure prediction methodology [29] has shown considerable progress in the related problem of predicting the structure of a protein based on the structure of a homologue [30]. The recently developed “rebuild and refinement” sampling methodology combines complete remodeling of the protein structure in specific regions [30] with global optimization of the entire protein structure using the Rosetta all-atom refinement protocol and energy function [29]. Because of the stochastic nature of the search, and the very large number of local minima on the rugged all-atom landscape, different models end up in different minima and these collectively create a map of the energy landscape in the neighborhood of the starting structure. Previously, this high-resolution refinement has been applied with the assumption that there is a single state to find, and it remains unclear whether the method has sufficiently high resolution to distinguish between two low-energy conformations in an allosteric protein.

Here we employ Rosetta to address the twin challenges of allostery: prediction and mechanism. We apply the high-resolution refinement method to the problem of finding an alternative conformation of a protein, which in this case represents the alternative allosteric state. Here we assume that multiple states exist, e.g. bound and unbound, and then ask whether Rosetta can identify the alternative state. We report that Rosetta can reproduce conformational transitions for three proteins in which significant allosteric structural changes occur, particularly when provided information on which regions change the most in the allosteric transition. Exploring the energy basins near each starting structure identifies state-dependent residues that control protein function. Mapping the energy minima suggests that energetically coupled residue pairs switch together in groups (blocks) that are weakly coupled to each other.

## Results

### Predicting the Alternative Conformation

We began by testing the extent to which the Rosetta high-resolution structure prediction methodology can predict the ligand free structure of an allosteric protein starting from the structure of the ligand bound form. We focus here on three allosteric proteins that undergo significant conformational changes upon effector binding: CheY, Integrin αL I-domain, and Ras. We initially selected 8 proteins (see Table 1) but restricted our efforts to these three proteins for the following reasons. Three of the others involved relatively small loop rearrangements induced directly by a ligand rather than global conformational changes induced by an allosteric effector. In the SH2 domain and FixJ, the energy difference between conformational states was too small for the Rosetta energy function to identify the correct conformation, while β-lactoglobulin involved a single loop difference where the deep energy minimum near the alternative structure wasn't sampled. The final two proteins, Troponin C and S100A6, involved calcium-binding sites for which the electrostatic interactions proved hard to model with the Rosetta energy function (Figure S1).

**Table 1:** Test set for predicting conformational change from bound to unbound state.

ID	L	Class	rmsd (Å) Xtal_unb_:Xtal_bnd_	rmsd (Å) Model:Xtal_bnd_	rmsd (Å) Model:Xtal_unb_	Protein name
1f4v, 3chy	128	α/β	1.22	1.33	0.91	CheY
1mq9, 1lfa	180	α/β	2.72	2.71	1.55	αL I-domain
6q21, 4q21	168	α/β	1.67	1.50	1.34	Ras p21
1lcj, 1bhh	104	α+β	1.53	1.17	1.59	SH2 domain
1b0o, 1beb	156	β	1.16	0.69	1.26	β-lactoglobulin
1d5w, 1dbw	123	α/β	1.25	2.24	2.15	FixJ
1avs, 1top	81	α	4.78	1.57	4.00	Troponin C
1k9k, 1k9p	82	α	4.23	2.44	3.41	S100A6

PDB IDs are given for the bound and unbound states in column 1. Protein length, SCOP classification and Cα-rmsd between crystal structures are given in columns 2 to 4. Cα-rmsd values between the lowest-energy model in the largest cluster and the bound and unbound crystal structures are given in columns 5 and 6 respectively.

**Figure 1:**
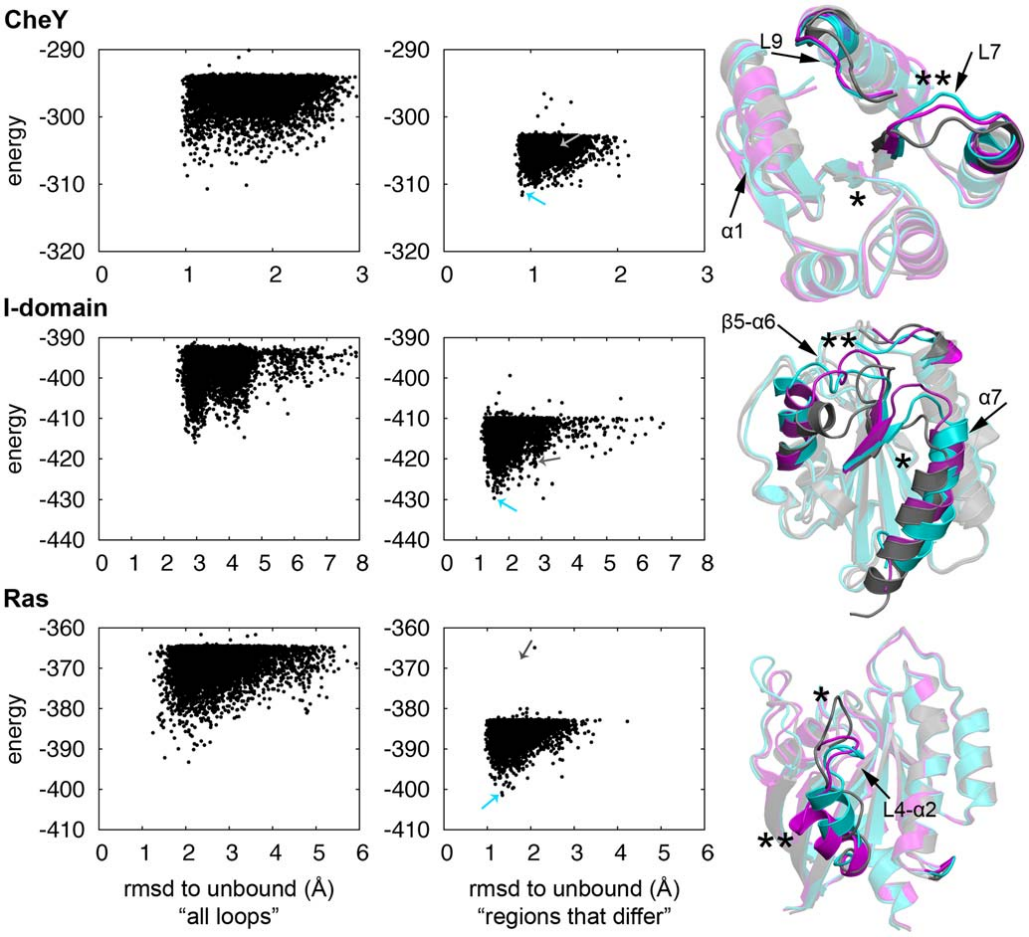
Rosetta predictions of conformational change in the allosteric proteins CheY, the αL I-domain, and Ras. The Rosetta all-atom energy is plotted against Cα-rmsd for models generated by simulations starting from the native conformation in the bound state with the allosteric effector removed from the crystal structure. Left panel shows the rmsd comparison to the alternative crystal structure when all loops have been remodeled, the center panel, the rmsd comparison to the alternative crystal structure with remodeling of loop or secondary structure regions that differ between the states. Arrows indicate the locations of the starting structure (gray) and lowest-energy model from the cluster with the largest number of structures (cyan). Right panel shows the superposition of the lowest-energy model taken from the largest cluster in the center panel (cyan) to the starting (gray) and alternative (magenta) crystal structures. The allosteric effector and protein binding site are indicated by * and ** respectively. The bright regions indicate regions that differ the most between the two crystal structures and were remodeled, while remaining regions are faded. Black arrows indicate the regions in the lowest-energy model that have moved toward the alternative state.

In this first set of calculations, all loop regions were stochastically rebuilt in the “rebuild” portion of the “rebuild and refinement” protocol described in ref [30]. 100,000 independent Monte Carlo “rebuilding and refinement” simulations were initiated from the bound conformations following removal of the ligand. Plots of energy vs root-mean-square deviation (rmsd) to the native structure (left panel of Figure 1) show that the deep energy minimum surrounding the native structure is sampled to some extent for Ras and CheY, as indicated by a minimum about 1 Å rmsd (the typical noise within a state) from the unbound state. However, this is not seen for the I-domain because regions with secondary structure differ in the two crystal conformations but were not allowed to be rebuilt in our initial calculations.

To make the sampling problem more tractable while modeling secondary structure movements, we limited the rebuilding step in the “rebuild and refinement” protocol to loop and secondary structure regions that significantly change structure in going from the bound to the unbound state (the entire protein is allowed to move in the following all-atom refinement step – see methods). The 20 lowest-energy structures were clustered based on their pairwise rmsd and the lowest-energy structure from the largest cluster was compared to both the starting and alternative structures. For three proteins (CheY, the αL I-domain, and Ras), the lowest-energy structure of the largest cluster was closer to the alternative conformation than the initial structure, and energy versus rmsd plots reveal an energy minimum at the unbound conformation (center panel of Figure 1). Additionally, the largest cluster of the 20 lowest-energy structures contained at least 4 models, suggesting that sampling is converging toward the alternative conformation. That is, with the specification of the regions in which major conformational changes take place, the rebuild and refinement protocol can sample the alternative state and the energy function has sufficient accuracy to distinguish the unbound state based on its lower energy.

In addition to identifying low-energy structures that are near the crystal conformation of the unbound state, subregions with the largest conformational difference between states were predicted to within an accuracy of between 0.3–3.4 Å (Cα-rmsd) to the alternative state (indicated by black arrows in Figure 1, and Table S1). The structural changes in CheY involve a shift of helix α1 and rearrangements of the loops L7 & L9 near the FliM binding pocket (indicated by **). Removal of a disulfide bond (allosteric effector indicated by *) in the αL I-domain that mimics the activated state allows the α7 helix to shift upward more than 6.5 Å and the loop between strand β5 and helix α6 to move toward the active conformation of the ICAM-1 binding site (indicated by **). In Ras, loop L4 moves away from the allosteric effector (located at *) and toward the alternative state, and the helix α2 near the protein-binding site (indicated by **) is formed, although it has not fully moved into position.

Because of high intrinsic variability in loop regions, we independently measured the RMSD only over the regular secondary structure elements, as described in Supplemental Table S2 and the accompanying description of the methods. In all three cases, the secondary structure elements were predicted on average even better than the overall structures, and the only regions of the secondary structure which remained closer to the starting structure than the alternative structure were those that differed very little between the states to begin with. Thus, Rosetta is most successful in predicting structural changes in secondary structure elements.

### Structural Differences

The crystal conformations of both states show a number of structural differences. Although many individual residues change conformation or contacts when an allosteric protein switches between states, only a small number of these changes may be critical to conformational switching [26]. To identify critical changes, we generated a set of 500 structures near each crystal structure by using Monte Carlo methods to perturb the backbone angles slightly and optimize side chain rotamer conformations, followed by energy minimization of each structure.

**Figure 2:**
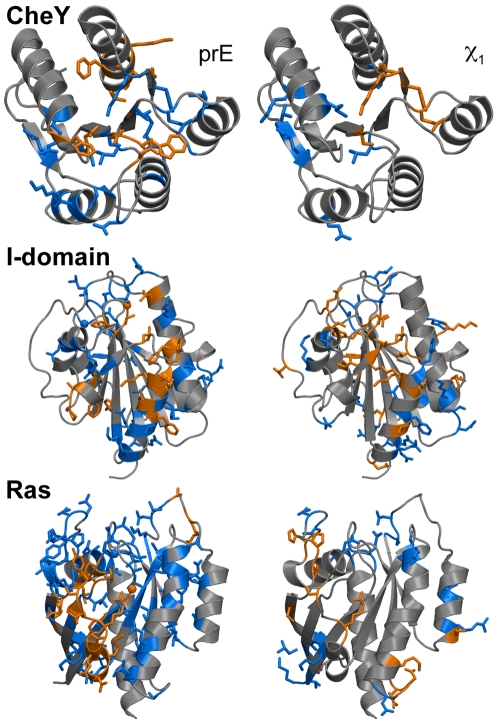
Contacts with large interaction energy differences or residues with large differences in their side chain dihedral angles. Differences between the crystal structures and the ensembles are mapped on to the three-dimensional structures of CheY, the αL I-domain, and Ras. Orange colored sticks indicate residues that make a state dependent contact (or have a state dependent side chain angle). Blue colored sticks reflect a contact (side chain) that differs between the two structures, but are not state dependent.

We first identified pairs of residues for which the mean difference in pairwise interaction energy (prE) was greater than 1 Rosetta energy unit between the 500 structures in the two ensembles surrounding each state. Since these contacts differ consistently between conformations in the two states, we call them “state dependent”. Averaging interaction energies over conformations in the two states eliminates the set of contacts that differ between the two structures not because of the change in conformational state but because of differences in crystal packing interactions. The left panel of Figure 2 shows the state dependent prE differences (orange) and the remaining (non state dependent) differences (blue) mapped on to the three-dimensional structure of CheY, the I-domain, and Ras.

CheY, the I-domain and Ras contain 128 to 180 residues, 27 to 82 of which formed pairwise interactions that had different energies in the two crystal structures. However, of these, only 10 to 20 formed state dependent interactions according to analysis of the ensemble of states (Table 2). Random mutagenesis [31]–[37] and mutations found in clinical samples [38]–[41] have identified a number of residues that alter protein function in the three proteins. Mutations identified by site-directed mutagenesis studies were not included since they are designed to target regions believed by the researchers to be important, which would cause an undesirable bias for our purpose. As shown in Table 2, there are a higher fraction of residues important for function among the residues with state dependent energy differences than in the protein as a whole. On average, using ensembles to identify state-dependent residues provided a 1.9-fold enrichment in the number of function-altering residues. A lesser (1.4-fold) enrichment was observed if the crystal structure differences were used to identify function-altering residues.

**Table 2:** Fraction of residues involved in pairwise interactions or side chain differences that are known to alter function.

	Pairwise Interaction	Side Chain χ_1_
	Whole Domain	Different in Crystal	State-Dependent in Ensemble	Whole Domain	Different in Crystal	State-Dependent in Ensemble
CheY	25/128 (20%)	7/27 (26%)	3/10 (30%)	22/102 (22%)	1/12 (8%)	1/5 (20%)
αL I-domain	44/180 (24%)	16/48 (33%)	10/17 (59%)	44/161 (27%)	11/39 (28%)	7/19 (37%)
Ras	51/168 (30%)	37/82 (45%)	11/20 (55%)	43/146 (29%)	12/29 (41%)	6/11 (55%)
Average	25 +/− 5%	35 +/− 10%	48 +/− 16%	25 +/− 4%	26 +/− 17%	37 +/− 17%
Enrichment	NA	1.4 +/− 0.1-fold	1.9 +/− 0.4-fold	NA	0.5 +/− 0.5-fold	1.4 +/− 0.5-fold

Columns 2 and 5 show this calculation for all residues, columns 3 and 6 for residues with significant differences in the crystal structures, and columns 4 and 7 for the residues with state-dependent differences in the ensembles.

We also identified state dependent side chain χ_1_ angles (dihedral angle rotation around the Cα–Cβ bond) based on mean differences in the χ_1_ angle between ensembles (right panel of Figure 2). Ensemble calculations identified 5, 19, and 11 residues (CheY, the I-domain, and Ras) with mean side-chain angle (χ_1_) differences greater than 46° between states (Table 2). Comparison between the calculated χ_1_ differences and the experimental data showed the state dependent residues contain a higher fraction of function-altering residues than in the protein overall (Table 2).

### Coupled Pairwise Changes

**Figure 3:**
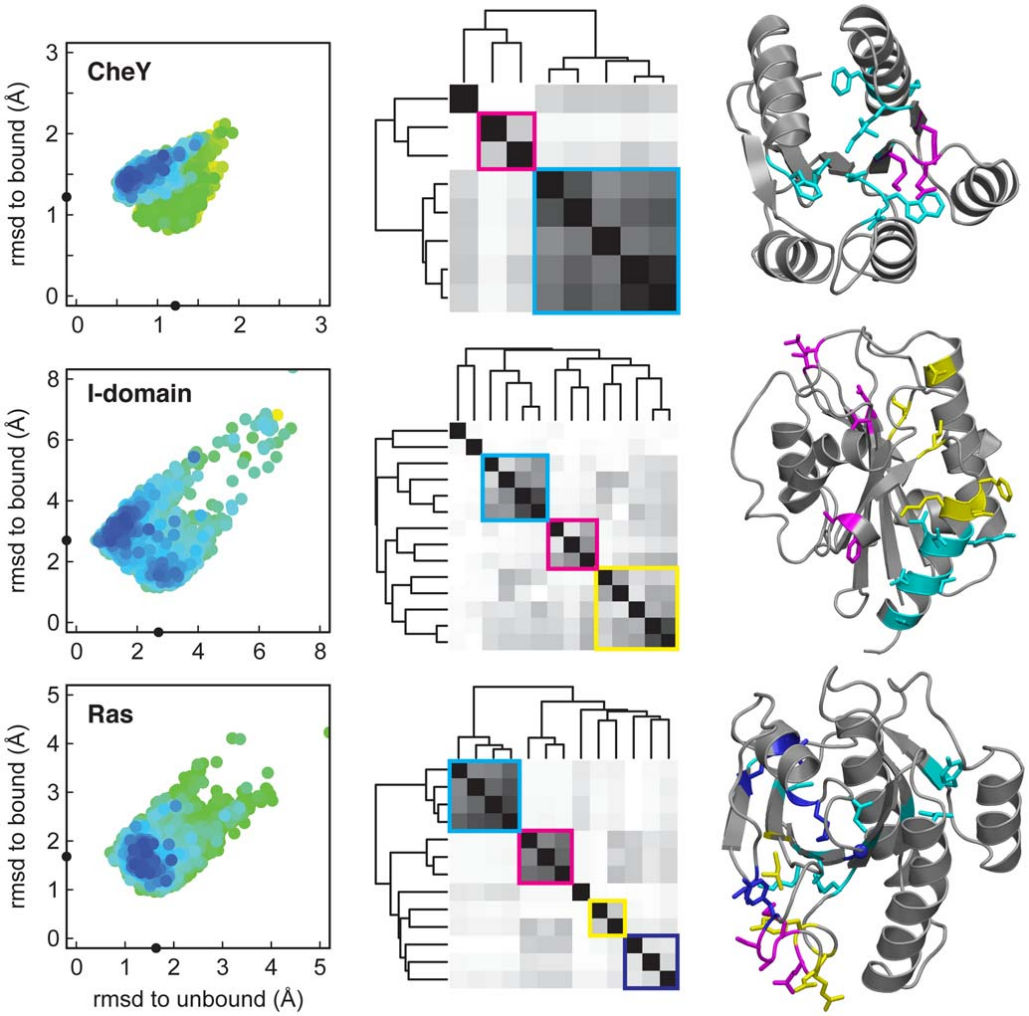
Residue-residue correlations in ensembles spanning the bound and unbound states. Left panel shows a two-dimensional map of the energy landscape showing the Cα-rmsd to the starting and alternative crystal conformations (locations are indicated by black dots on the axes). Each point represents a single low-energy model on the landscape colored by energy from low (blue) to high (red). The central panel shows a hierarchical clustering of the association (ϕ coefficient) between pairwise interactions. The white to black coloring reflects the association between residue pairs, where white represents no association (ϕ = 0) and black represents a strong association (ϕ = 1). A colored square has been added around strongly associated clusters of pairwise interactions. The right panel maps the residue pairs with the strongest associations onto the three-dimensional protein structure. Residues are colored based on the hierarchical clustering.

To examine how pairwise interactions are coupled during switching between the states, we generated models starting from the unbound state to map the neighboring landscape more thoroughly. Maps of the energy landscapes for CheY, the I-domain and Ras were created by combining the “rebuild and refinement” calculations starting from the bound and unbound structures (left panel of Figure 3). Each point on this landscape represents a single model, the axes are the rmsd values to the starting and alternative structures, and the colors represent the all-atom energy, graded on a continuum from lowest (blue) to highest (red). A clear minimum is evident in the vicinity of the unbound state in all three cases, as indicated by a cluster of low-energy structures near 1 Å rmsd from the unbound state and over 1 Å rmsd from the bound state. Each structure on this landscape represents a distinct local minimum—the lowest energy structure sampled in an individual simulation.

The two-dimensional view of the energy landscape suggests we have sampled the conformational space of both states and have reasonable coverage of intermediate conformations. To determine what residues switch conformational states together, we evaluated the association between pairwise contacts (see methods). Some residues are strongly correlated and evidently switch states together whereas others switch independently. The correlated pairwise interactions appear as blocks when grouped using hierarchical clustering (middle panel of Figure 3). Within a block, the pairwise interactions show a stronger association than between blocks. In the context of the three-dimensional protein structure, the blocks comprise collections of residues that are often physically close to one another (right panel of Figure 3).

Different blocks are often associated with different functions. In CheY, the cyan block includes highly conserved amino acids (D12, D57, and K109) involved in phosphorylation and regulation of this receiver domain [42],[43]. The magenta block contains residues E89 and Y106, which play critical roles in conformational switching through CheZ-mediated dephosphorylation [44] and binding to the flagellar motor switch, FliM [45],[46]. These two blocks are also related to functional regions observed in a previous study of internal dynamics with NMR [47].

In the αL I-domain, the blocks of coupled residues divide into three groups, which roughly map out a connection path between helix α7 (cyan) and the ICAM-1 binding site (residues within the magenta block such as D127 & L205). The yellow block that connects these regions includes residues from the β6-α7 loop and the hydrophobic pocket proposed to be responsible for the ratchet-like conformational switching [48].

In Ras, the magenta and yellow colored blocks contain residues in the helical-loop segment known as switch II [49], which is directly involved in conformational switching between the active and inactive states. The cyan colored block contains contact pairs within the hydrophobic core that is highly conserved among Ras family proteins. This block is comprised of a set of coupled pairs that span the core β-sheet, connecting one side of the protein to the other.

## Discussion

### Predicting the Unbound/Inactive Conformation

Using the Rosetta rebuild and refinement sampling methods, the bound states of three allosteric proteins were observed to relax to the lower energy unbound states. Accurate prediction of the unbound state is facilitated by focusing sampling on the loops and secondary structure regions that differ between states. The Rosetta energy function is able to identify the correct structure; the need to focus the rebuilding protocol on regions known to differ is consistent with previous observations that conformational sampling is the primary limiting factor in high-resolution prediction. Nevertheless, our successes provide evidence of useful progress toward predicting conformational changes in allosteric proteins when only the bound structure is available. These successes are indicated by a decrease in the overall Cα-rmsd between the low-energy model and the alternative state, as well as a substantial improvement in the Cα-rmsd between the low-energy model and the alternative state for the subregions that differ most between states (Table S1).

The sampling strategy failed to explore conformational space near the alternative state in proteins with large conformational changes that involved the hinge motion of multiple helices. The Rosetta energy function is insufficiently accurate to identify the correct structure for proteins with subtle loop changes where the energy difference between states is likely quite small, or those with electrostatic interactions with divalent cations. These challenges emphasize the need for improvements in both the Rosetta energy function and sampling strategies for exploring conformational space. However, since predicting the unknown conformation of an alternative state remains an unsolved problem, even partial success in this direction is encouraging and suggests that this approach warrants further development.

### Structural Differences

We calculated the mean differences between pairwise interactions and side chain χ_1_ angles in ensembles of low-energy models near each state. These calculations provide a way to screen *in silico* a large number of conformational differences to identify a smaller set of promising residues to target for further experimental investigation. As indicated by random mutagenesis and mutations found in clinical samples, the state-dependent residues are enriched in amino acids known to control function (Table 2). The positive correlation between predictions and experiments suggests that ensembles could be used to predict state-dependent residues to mutate in order to alter the regulation of conformational switching. For example, it may be possible to change the overall activity but not the specificity of a protein by mutating state-dependent residues that are not in either effector or active sites, but rather in the pathway between them.

### Coupled Pairwise Changes

**Figure 4:**
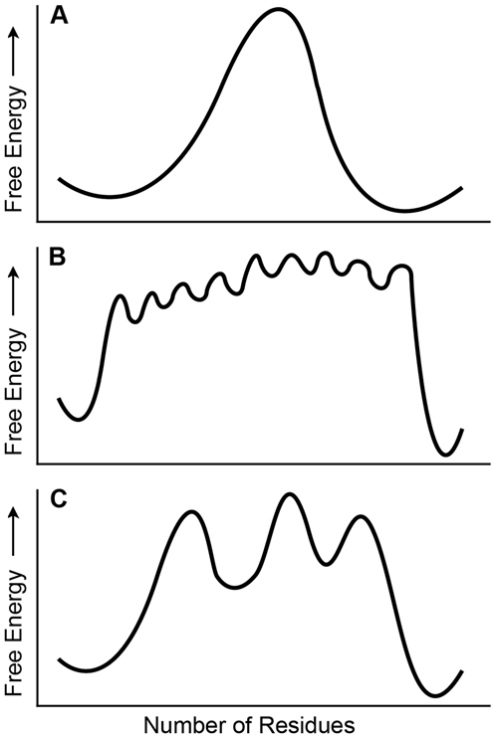
Conformational switching models in allosteric proteins. Schematic energy profile plotted as a function of the number of residues that change between states on a one-dimensional energy landscape. (A) All-or-nothing model in which all residues switch together and the conformational change between states happens in a concerted manner. (B) Domino model in which one residue interacts with its neighbor and so on as the conformational change between states proceeds along a specific propagation pathway. (C) Block model in which groups of tightly coupled interactions switch together and each block is loosely coupled to other blocks such that conformational change between states happens through interacting blocks. All of these models would appear as two-state transitions experimentally, however, the domino and block models transition through multiple intermediate states.

The state dependent contact pairs group into clusters (blocks) that are often nearby on the three-dimensional structure and correlated with specific functions. These clusters of residue pairs tend to switch together in conformations spread throughout the energy landscape between the starting and alternative states. Each switching group maintains a weak association to other blocks of residue pairs, and these blocks form a weakly coupled system that could pass information between more distance regions of a protein. We propose a new “block” model (Figure 4C) for allosteric transitions that is intermediate between a concerted model, where all structural changes are tightly coupled and conformational switching is completely cooperative (Figure 4A), and a sequential or domino model, where binding of a molecule at one site causes a sequential propagation of changes across the protein in a defined pathway (Figure 4B). This suggestion is conceptually similar to the previous suggestion, based on dynamics simulations, that protein conformational changes [20], including those the occur due to ligand binding [21], can occur via a pathway that involves multiple basins. Because the two methods have been applied to different proteins, and because the data that suggests multiple intermediates is of a different nature, however, it is difficult to compare the details of the proposed intermediates.

The high-energy states of all three models in Figure 4 are not readily observed experimentally. However, our model suggests that stabilizing the energetically coupled residues in one conformational state would lower the energy of that intermediate state to the point where it might be observed. The block model is physically plausible in that sets of residues that pack together would be expected to be highly correlated and switch states cooperatively, while more weakly coupled to residue clusters at distant sites. Allostery in this model is a result of the (weak) coupling between clusters of tightly interacting residues: a switch in state at a first cluster alters the energetic balance between alternative states at other clusters.

Our approach differs in both methodology and conclusions from previous computational methods of studying allostery [11]–[13],[22],[23],[50]. It is particularly instructive to compare our approach to previous work using all-atom molecular dynamics. A clear disadvantage of our method is that since we do not simulate dynamics, we can obtain no explicit information about trajectories, dynamics, or kinetics. We cannot observe pathways directly. On the other hand, our approach has two clear advantages. First, each data point is from a completely independent Monte Carlo Minimization simulation, hence observed correlations between contacts and other properties cannot be attributed to lack of independence in sampling (as might be the case for different snapshots from a long MD trajectory). Second, each data point represents a relatively deep local minimum (the lowest energy point found in the MCM simulation), and hence associations between residues may be stronger than in higher energy states—the higher the energy, the larger the noise due to energy fluctuations. Our approach focuses on the energetic coupling between interactions in allosteric transitions rather than the dynamic coupling.

## Methods

### Test Set Selection and Starting Model Preparation

To test whether it is possible to predict a ligand-induced conformational change in allosteric and non-allosteric proteins, we selected a set of 8 pairs of ligand bound and ligand free protein structures from the Protein Data Bank [51] (Table 1). Coordinates for the starting structure of the αL I-domain were modified according to [52]. The selection criteria were the availability of structures of ligand bound and ligand free forms, a significant structural rearrangement (Cα-Cα differences >3.5 Å) between the two forms, and size less than 200 amino acids to ensure the tractability of the search problem. All crystal structures had a resolution ≤ 2.5 Å, and with the exception of three bound structures (PDB ID: 1b0o, 1f4v, 1d5w) the structures were ≤ 2.0 Å resolution.

Test cases were grouped into categories based on their conformational change and their structural classification (all-α, all-β, mixed α/β or α+β) [53]. These categories allowed us to evaluate the method's ability to predict both localized and allosteric conformational changes with high-resolution accuracy, as well as to consider how a protein's fold affected the conformational sampling and prediction accuracy. Starting models were created from the crystal structures by fixing the bond lengths and angles at chemically ideal values, and representing all atoms explicitly using internal coordinates (ϕ, ψ, ω, χ_1_, χ_2_, χ_3_, & χ_4_). Following idealization, all models were minimized as a function of all backbone and side chain angles using the Davidon-Fletcher-Powell (DFP) algorithm [54].

### Prediction Protocol

The structure prediction protocol is based on the “rebuild and refinement” method that is outlined in detail elsewhere [30]. Briefly, the overall approach consisted of three parts, (1) generating structural diversity, (2) optimizing the side chain position for every residue, and (3) minimizing all atoms in the protein. In the rebuild step, structural diversity was created by replacing backbone torsion angles of the loops with one or three or nine consecutive residues “fragments” from non-homologous structures in the Protein Data Bank. Initially, all loop regions were remodeled. Based on insufficient sampling of the conformational space near the alternative structure, we then chose to rebuild continuous sequences of 4 or more residues where the pairwise Cα-Cα difference was greater than 1 Å (>1.5 Å for Troponin C and S100A6). These chosen regions were randomly selected during a simulation to be remodeled using the fragment insertion protocol as described in [55]. Briefly, a chain break (“cut”) was made to the remodeled segment at a randomly chosen position within the region. Randomly chosen nine-residue, three-residue, or one-residue fragments were inserted into randomly chosen positions in the region being rebuilt, and the Metropolis Monte Carlo criterion was used to accept or reject the newly inserted fragment. To maintain the connectivity of the protein chain, cyclic coordinate descent [56] was used to close the chain break at a stochastically selected position of the region rebuilt.

In the refinement protocol, all of the backbone and side chain atoms in the protein are explicitly represented. The entire protein is allowed to move through a series of steps that introduce a random perturbation to the backbone atoms, and then optimize the backbone and side chain coordinates for the new backbone position (see [30] for a detailed description of the types of random perturbations and the move sequences). Optimal side chain conformations for each residue were selected from the Dunbrack rotamer library [57]. After the backbone perturbation and side chain optimization, the energy of the entire structure was minimized as a function of all backbone and side chain dihedral angles using the DFP algorithm. The new angles were accepted or rejected using the standard Metropolis criterion between the energy of the minimized structure and the initial conformation prior to the random perturbation. This entire cycle of rebuild and refinement was repeated ∼100,000×, generating ∼100,000 low-energy conformations of each protein in the test set, and exploring a broad set of local minima within the energy landscape that are both near and far from the starting conformation.

### Clustering Algorithm

The top 20 low-energy models were selected from the set of ∼100,000 simulations and clustered based on a structural similarity using an algorithm that has been described previously [58]. Briefly, pairwise Cα-rmsd comparisons were made between all 20 models using a threshold of 1.0 Å to define neighboring structures. The structure with the largest number of neighbors within this threshold was considered to be the center of the first, largest cluster. This cluster center and its neighbors were removed from the population and the pairwise comparison was repeated until all structures in the set were examined. The lowest-energy structure in the cluster with the largest number of neighbors was selected for comparison to the starting and alternative crystal structures.

### Near-Native Ensemble Generation

The crystal structure was taken as the starting template for creating an ensemble of near-native models. Bond lengths and angles were fixed at ideal values and each structure was minimized. Following idealization and minimization, all proteins within the test set were subjected to the Monte Carlo plus minimization (MCM) protocol to generate 500 models in the vicinity of the crystal conformation. The MCM strategy uses the all-atom, high-resolution refinement protocol that has been described previously [29],[59]. Briefly, the MCM strategy consists of small, random perturbations to the backbone torsion angles, optimization of the side-chain rotamer conformations for the new backbone angles, and minimization of the backbone and side chain degrees of freedom using the DFP algorithm.

### Pairwise Interaction Energy Changes and Side Chain Differences

The pairwise interaction energy (prE) was computed from a subset of terms in the Rosetta energy function including the Lennard-Jones attractive and repulsive, hydrogen bonding, solvation, and a statistical term (“pair”) that approximates electrostatics and disulfide bonds, 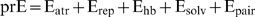. Mean prE differences greater than 1 Rosetta energy unit between the ensembles of 500 near-native models were considered to be state dependent.

The χ_1_ side-chain angle (dihedral rotation about Cα–Cβ bond) was computed for all residues except alanine and glycine. Mean χ_1_ differences [60] greater than 46° [61] between the ensembles of 500 near-native models were considered to be state dependent.

State-dependent predictions were compared against residues that have been experimentally found to alter protein function by random mutagenesis, or mutations found in clinical samples. Function-altering mutations identified by site-directed mutagenesis studies were excluded since they are designed to target regions believed by the researchers to be important, which would cause an undesirable bias for our purpose. The fraction of residues involved in either pairwise interactions or side chain differences that are known to alter protein function was computed for the whole protein (*f_tot_*), the differences in the crystal structures (*f_xtal_*), and the state-dependent residues in the ensembles (*f_ens_*). The ratio of fractions (*f_xtal_*/*f_tot_* and *f_ens_*/*f_tot_*) was calculated to determine the enrichment of function-altering residues present in the computed differences versus the whole protein.

### Evaluation of Pairwise Energy Coupling

The pairwise interaction energy (as described above) was computed for all residue pairs in both states of CheY, the αL I-domain, and Ras. Pairwise coupling was evaluated by examining the pairs that changed contact between states. These changes were considered to be binary and involved going from interacting (prE <–1.25 Rosetta energy units) to non-interacting (prE >−0.5 Rosetta energy units). Calculations were performed on all models from the two sets generated by starting from the bound and unbound states and running the “rebuild and refinement” protocol to explore the neighboring energy landscape.

Associations between pairwise interactions were computed from the φ coefficient, where 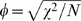. χ^2^ is the chi-square statistic for testing independence (
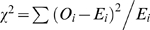
, where *O* and *E* are the observed and expected frequency) and *N* is the number of observations. Associations were clustered using the complete-linkage, hierarchical clustering algorithm implemented in the R statistical package (http://www.r-project.org/).

### Software

All plots were made with gnuplot (http://www.gnuplot.info/) or the R statistical package (http://www.r-project.org/). Images of protein structures were generated using PyMOL [62]. The Rosetta source code is available without charge for academic users from http://depts.washington.edu/ventures/UW_Technology/Express_Licenses/rosetta.php

## Supporting Information

Figure S1 Rosetta Calculations of Conformational Change for Remaining Proteins in Test Set. All-atom energy is plotted against Cα-rmsd for models generated by simulations starting from the native conformation in the bound state with the ligand removed from the crystal structure. Left panel shows the rmsd comparison to the alternative crystal structure when all loops have been remodeled, whereas the center panel shows the rmsd comparison to the alternative crystal structure with only remodeling regions that differ between the states. Right panel shows the superimposition of the starting (gray) and alternative (magenta) crystal structures. Corresponding plots for CheY, the αL I-domain, and Ras are presented in Figure 1.
(2.27 MB TIF)

Table S1 Comparison between subregions that change most between conformational states
(0.03 MB DOC)

Table S2 Description of rmsd calculations on secondary structure elements-sheets and helices-for CheY, αL I-domain, and Ras.
(0.06 MB PDF)
