# Supplementary figures and images for: Computation of Conformational Coupling in Allosteric Proteins
Source: PLoS Comput Biol. 2009 Aug 28;5(8):e1000484. doi: 10.1371/journal.pcbi.1000484 (PMC2720451; doi:10.1371/journal.pcbi.1000484)

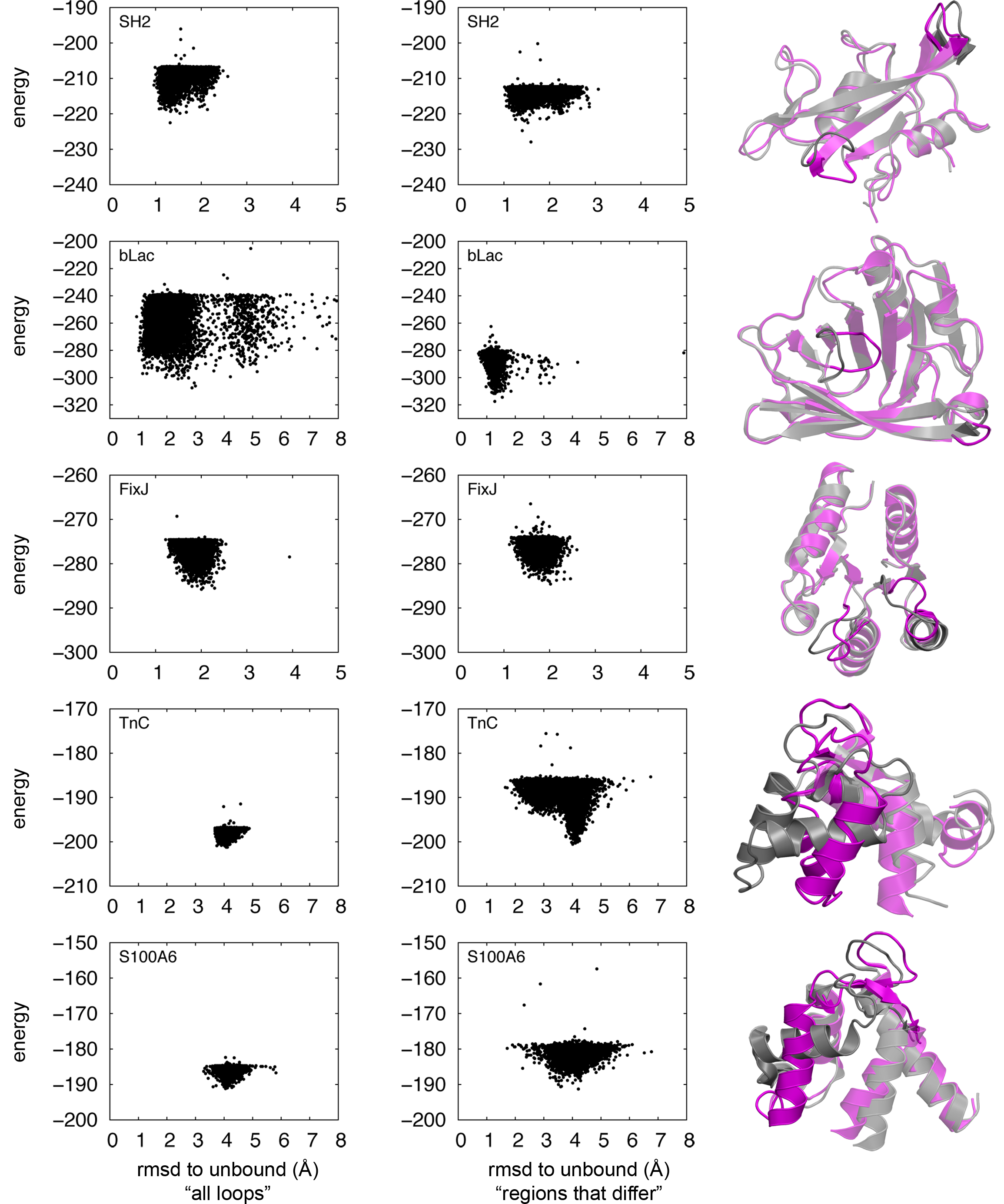

Supplement: Figure S1 — Rosetta Calculations of Conformational Change for Remaining Proteins in Test Set. All-atom energy is plotted against Cα-rmsd for models generated by simulations starting from the native conformation in the bound state with the ligand removed from the crystal structure. Left panel shows the rmsd comparison to the alternative crystal structure when all loops have been remodeled, whereas the center panel shows the rmsd comparison to the alternative crystal structure with only remodeling regions that differ between the states. Right panel shows the superimposition of the starting (gray) and alternative (magenta) crystal structures. Corresponding plots for CheY, the αL I-domain, and Ras are presented in Figure 1. (2.27 MB TIF) [file pcbi.1000484.s001.tif]
