# Supplementary material for: Computation of Conformational Coupling in Allosteric Proteins
Source: PLoS Comput Biol. 2009 Aug 28;5(8):e1000484. doi: 10.1371/journal.pcbi.1000484 (PMC2720451; doi:10.1371/journal.pcbi.1000484)
Supplement: Table S1 — Comparison between subregions that change most between conformational states (0.03 MB DOC) [file pcbi.1000484.s002.doc]

**Table S1. Comparison between subregions that change most between conformational states.**

| Protein | Region | Difference Between Crystal Structures | Difference Between Low-energy Model and Alternative State |
| --- | --- | --- | --- |
| CheY | α1 (14–29) | 1.0 Å | 0.3 Å |
|  | Loop 7 (88–92) | 2.2 Å | 1.3 Å |
|  | Loop 9 (109–113) | 1.9 Å | 0.5 Å |
|  |  |  |  |
| αL I-domain | β5-α6 (261–275) | 3.7 Å | 2.6 Å |
|  | α7 (293–308) | 6.0 Å | 2.7 Å |
|  |  |  |  |
| Ras | Switch II (59–76) | 4.4 Å | 2.9 Å |
|  | Loop 4 (59–65) | 5.4 Å | 3.4 Å |
|  | α2 (66–76) | 3.7 Å | 2.4 Å |

Cα-rmsd between stretches of subregions, indicated in parentheses, after alignment over all Cα atoms.
