# Supplementary material for: Computation of Conformational Coupling in Allosteric Proteins
Source: PLoS Comput Biol. 2009 Aug 28;5(8):e1000484. doi: 10.1371/journal.pcbi.1000484 (PMC2720451; doi:10.1371/journal.pcbi.1000484)
Supplement: Table S2 — Description of rmsd calculations on secondary structure elements-sheets and helices-for CheY, αL I-domain, and Ras. (0.06 MB PDF) [file pcbi.1000484.s003.pdf]

Description of rmsd calculations on secondary structure elements—sheets and helices—for CheY, αL I-domain, and Ras

Methods

Secondary structure calculations were performed using DSSP [1]. Strands and helices were selected based on residue sequences ($n > 2$) with strand (code is E or B) or helical (code is H or G) propensities. RMSD calculations were performed using VMD [2]. The structures were aligned using all Cα-atoms—with the alternative structure as the reference—prior to any RMSD comparisons between secondary structures regions.

Results

The overall trend shows that the Rosetta model is closer to the alternative crystal structure for almost every secondary structure element (this is certainly true for all secondary structure elements with larger differences between crystal structures). This result is particularly important because we did not explicitly remodel the majority of these regions when running the Rosetta predictions. Instead, the all-atom refinement stage (stage where these regions are allowed to move) moved these regions into alignment with the alternative crystal structure. Table S2 summarizes the RMSD differences between secondary structure regions for each protein (M = prediction, X_A = alternative crystal structure, X_S = starting crystal structure). Numbers highlighted in red are the regions where the predicted model was not closer to the alternative structure than the two crystal structures (only a handful of regions). In general, this situation occurs when the difference between the crystal structures is very small (< 0.7 Å).

Table S2. Comparison between secondary structure subregions.

**CheY**			**αL I-domain**			**Ras**		
Region	M:X_A (Å)	X_A:X_S (Å)	Region	M:X_A (Å)	X_A:X_S (Å)	Region	M:X_A (Å)	X_A:X_S (Å)
All Cα	0.91	1.22	All Cα	1.55	2.72	All Cα	1.34	1.67
All SS	0.54	1.22	All SS	1.08	2.42	All SS	1.02	1.36
β1: 7–10	0.34	0.21	β1: 2–9	0.50	1.17	β1: 2–10	0.49	0.63
β2: 32–35	0.47	0.20	β2: 38–45	0.51	1.06	β2: 37–46	0.75	1.13
β3: 52–55	0.23	0.32	β3: 49–53	0.55	0.71	β3: 49–58	0.82	0.82
β4: 82–86	0.34	0.90	β4: 103–110	0.46	1.16	β4: 77–83	0.50	0.44
β5: 104–107	0.37	0.38	β5: 127–132	0.77	1.27	β5: 111–116	0.38	0.35
			β6: 158–160	1.11	2.56	β6: 141–145	0.26	0.45
α1: 14–27	0.29	1.09						
α2: 38–46	0.52	0.28	α1: 16–32	1.06	2.32	α1: 16–23	0.68	0.35
α3: 64–73	0.34	0.45	α2: 55–61	1.03	1.43	α2: 66–74	2.69	4.04
α4: 91–99	0.72	1.18	α3: 64–67	0.68	1.13	α3: 87–103	0.80	1.04
α5: 112–127	0.81	2.24	α4: 80–90	0.68	0.59	α4: 127–137	0.83	0.68
			α5: 144–149	1.63	1.93	α5: 152–167	0.66	0.41
			α6: 154–157	1.31	1.78			
			α7: 166–176	2.05	5.95			

References

1. Kabsch W, Sander C (1983) Dictionary of protein secondary structure: pattern recognition of hydrogen-bonded and geometrical features. Biopolymers 22:2577–2637.

2. Humphrey W, Dalke A, Schulten K (1996) Vmd: Visual molecular dynamics. J Mol Graph 14:33–38.
